# Considering the design effect for the stepped wedge trial: what can it tell us?

**DOI:** 10.1186/1745-6215-16-S2-O45

**Published:** 2015-11-16

**Authors:** Jennifer Thompson, Katherine Fielding, James Hargreaves, Andrew Copas

**Affiliations:** 1 Department of Infectious Disease Epidemiology, London School of Hygiene and Tropical Medicine, London, UK; 2 Department of Social and Environmental Health Research, London School of Hygiene and Tropical Medicine, London, UK; 3 MRC London Hub for Trials Methodology Research, MRC Clinical Trials Unit at University College London, London, UK

## 

In stepped wedge cluster randomised trials (SWTs) the intervention is rolled out to clusters in a random order. Measurements are collected from different individuals during each step (the time between clusters switching to intervention). It remains unclear how to optimise power in SWTs and the circumstances in which SWTs requires fewer clusters to achieve the same power as parallel cluster randomised trials (CRTs) or CRTs with half of all measurements at baseline (CRT-Bs).

We consider the design effect for SWTs in terms of the number of measurements per cluster N to investigate how inclusion of baseline measurements in N, and number of steps influence the number of clusters required for a given power. We compared the design effects for SWTs, CRTs and CRT-Bs holding N constant.

For SWTs, the number of clusters required is increased by including baseline measurements. Increasing the number of steps reduces the number of clusters, with less marginal effect as the number of steps increases. SWTs with no baseline measurements and many steps require fewer clusters than CRTs when the intracluster correlation ICC> 1/(N+1). CRT-Bs always require more clusters than SWTs with no baseline measurements and more than two steps. A CRT-B and two-step SWT are equivalent designs.

To minimise number of clusters, SWTs should exclude baseline measurements. The reduction in number of clusters for SWTs compared to CRTs is greatest when N is large (e.g. N=100) and ICC is high (e.g. ICC=0.2). SWTs however require a more careful analysis to remove confounding by secular trends.

